# Body mass index and weight change during initial period of chemotherapy affect survival outcome in advanced biliary tract cancer patients

**DOI:** 10.1371/journal.pone.0195118

**Published:** 2018-04-02

**Authors:** Jinwoo Kang, Sang Hyub Lee, Jun Hyuk Son, Jae Woo Lee, Young Hoon Choi, Jin Ho Choi, Woo Hyun Paik, Ji Kon Ryu, Yong-Tae Kim

**Affiliations:** 1 Department of Internal Medicine and Liver Research Institute, Seoul National University College of Medicine, Seoul, Korea; 2 Department of Internal Medicine, Seoul National University Hospital, Seoul, Korea; 3 Department of Gastroenterolgy and Hepatology, SMG-SNU Boramae Medical Center, Seoul, Korea; 4 Department of Internal Medicine, Inje University Ilsan Paik Hospital, Goyang, Korea; 5 Mediplex Sejong Hospital, Gyeyang-gu, Incheon, Korea; Texas A&M University, UNITED STATES

## Abstract

**Background:**

The impact of obesity on survival is known to vary in different cancers. Advanced biliary tract cancer was rarely analyzed about the relationship between obesity and prognosis. We performed this study to evaluate the BMI and body weight change as prognostic factors for advanced biliary tract cancer patients with palliative chemotherapy.

**Methods:**

Between January 2005 and December 2016, two hundred and seventy-six patients who underwent chemotherapy for biliary tract cancer were retrospectively analyzed. The relationship between BMI (kg/m^2^) and clinical outcomes including overall and progression-free survival was assessed. Additionally the relationship between change in body composition and overall survival was evaluated.

**Results:**

Median overall survival was 9.7 months for underweight patients, 10.1 months for normal patients, 15.8 months for overweight group, 13.1 months for obese patients, respectively. (p = 0.047) Univariate analysis showed that BMI, stage III, age less than 64 year-old, gallbladder cancer, operation, radiotherapy and ECOG performance were significantly associated with better survival. Compared with normal patients, overweight patients (BMI 23–24.9kg/m^2^) had a reduced risk of mortality in multivariate analysis (HR 0.632; 95% CI 0.436–0.918, p = 0.016). In the additional analysis for the effect of changes in body weight and BMI to the overall survival, decrease in body weight and BMI (HR 1.410, 95% CI 1.168–1.986, p = 0.046) was associated with a shorter in overall survival.

**Conclusion:**

Overweight status and the maintenance of body weight during the initial period of chemotherapy are important and independent predictors of better overall survival in advanced biliary tract cancer patients.

## Introduction

Biliary tract cancer (BTC) is a rare but fatal neoplasm that arises from biliary epithelium. Biliary tract cancer consists of gallbladder cancer and cholangiocarcinoma categorized as an intrahepatic duct, hilar, and distal bile duct tumor. Prognoses of both gallbladder cancer and cholangiocarcinoma are known to be very poor. The median survival of patients with unresectable biliary tract cancer is reportedly 3 to 6 months with or without biliary drainage [1].

The prevalence of obesity has increased markedly over the past few decades, and obesity has become a major burden on global public health, especially in developed countries. Obesity is considered a major risk factor for the progression of many chronic diseases. However, many studies have demonstrated that obesity is subject to the so called ‘Obesity Paradox,’ in which it acts a protective factor in patients with chronic diseases such as cardiovascular, chronic kidney disease, chronic obstructive pulmonary disease, and rheumatoid arthritis [2].

Numerous researches have investigated the factors affecting clinical outcomes of the patients with cancer. In studies investigating the association of body mass index (BMI) with clinical outcomes in many kinds of cancer patients, an increased BMI has been associated with mixed outcomes [3, 4].

Few studies have investigated the BMI in patients with biliary tract cancer, and most of these evaluated the association of BMI and the risk of developing cancer. Many studies have reported that obesity increased the risk of developing biliary tract cancer [5–7]. In a study published in 2003, mortality increased with increasing BMI among patients with gallbladder cancer [8]. Conversely, in a study with 55 cholangiocarcinoma patients, there were no significant differences between BMI and survival outcome [9]. However, this was a small-scale study of patients undergoing surgery. Therefore, the results of these studies cannot be applied directly to unresectable BTC, which accounts for most BTCs. In addition, no studies have evaluated BMI and body weight changes as prognostic factors in patients who underwent chemotherapy, which is the mainstay of treatment for unresectable BTCs.

Taking these points into consideration, we evaluated the association between BMI and clinical outcomes including overall and progression-free survival and whether body weight changes can be a prognostic factor for advanced BTC patients receiving palliative chemotherapy.

## Methods

### Study subjects and data collection

We retrospectively reviewed the medical records of 425 patients who had been diagnosed with advanced biliary tract cancer and underwent palliative chemotherapy between January 2005 and December 2016. All subjects were diagnosed with CCA on the basis of histological or cytological confirmation.

The following data were obtained for each patient: age, sex, BMI, comorbidities, Eastern Cooperative Oncology Group Performance Status (ECOG-PS), tumor location, tumor stage, existence of distant metastases, regimen of chemotherapy. Patients were only included if they were at least AJCC tumor stage III, ECOG-PS ≤2, Charlson comorbidity index ≤5, had no history of other types of cancer, were previously untreated with palliative chemotherapy, had undergone at least three cycles of chemotherapy, and did not have active biliary infection at the time of chemotherapy.

CT scans performed at the time of initial chemotherapy and 2-month follow-up were used to quantify skeletal muscle mass. Two adjacent axial images within the same series were selected for the analysis of the third lumbar vertebra (L3) skeletal muscle and the mean of the 2 measurements was calculated. Muscles were quantified within a Hounsfield unit (HU) range of -29 to 150 HU [10]. Muscle mass was normalized for height in meters squared(m^2^) and reported as the lumbar skeletal muscle index (SMI; cm^2^/m^2^) [11].

Overall, 276 patients were analyzed in this study. All patients were followed until December 2016, and observations were censored at the time of death or loss to follow-up. The study protocol was approved by the institutional review board of Seoul National University Hospital. All studies were conducted according to Declaration of Helsinki. Requirement for informed consent was waived for this retrospective study and all patients’ medical records were fully anonymized before analysis.

### Definition of the variables

In our hospital, we conducted laboratory tests and measured patients’ height and weight for dose adjustment on the first day of each chemotherapy cycle. These data were also used to determine the BMI. In this study, BMI measured at the start of chemotherapy was used for classification based on the WHO classification, Asia-Pacific guidelines of obesity classification and referring to the clinical practice guidelines for overweight and obesity in Korea: underweight (BMI <18.5kg/m^2^), normal weight (BMI: 18.5–22.9kg/m^2^), overweight (23–24.9kg/m^2^), and obese (≧25kg/m^2^). [12] Changes in body weight were categorized as follows: -5% to 5% (no change), -10% to -5%, more than -10%, 5% to 10%, and more than 10%. Sarcopenia was defined as SMI ≤41cm^2^/m^2^ based on previous studies [11, 13].

Tumor location was classified as gallbladder cancer or bile duct cancer, and the latter was categorized into intrahepatic, proximal bile duct (including Klatskin tumor), and distal bile duct. The stage of the tumor was recorded according to the AJCC TNM staging classification (7^th^ ed.). Progression-free survival (PFS) was defined as the time interval from the start of the palliative chemotherapy to disease progression, and overall survival (OS) was defined as the time from start of palliative chemotherapy to death from any cause or last day of follow-up.

### Statistical analysis

Data are shown as the number (%) for categorical variables, the mean ± standard deviation (SD) for continuous variables and the median for survival. The significance of differences in clinical parameters among BMI groups was assessed by the Chi-squared test, one-way analysis of variance (ANOVA), and Fisher’s extract test as appropriate. Overall, survival and progression-free survival curves were constructed by the Kaplan-Meier method and compared using the log rank test. A multivariable Cox proportional hazards model was used to identify the independent predictors of PFS and OS. Results were expressed as hazard ratios (HRs) and 95% confidence intervals (CIs). Statistical significance was assumed at a confidence level of 0.05. Statistical analyses were performed using SPSS 23 (SPSS, Chicago, IL, USA).

## Results

### Baseline characteristics of patients

Baseline characteristics of the 276 patients are summarized in Table 1. There were 177 male and 99 female patients aged 26 to 94 years (63.7 ± 9.9). Median survival of the patients was 11.1 months (range: 1.5–71.6, 11.1±10.3). Of the 276 patients included in this study, 30 (10.9%) were underweight, while 113 (40.9%), 64 (23.2%), and 69 (25.0%) patients were classified as normal, overweight, and obese, respectively. The mean SMI value was 43.3±15.5 cm^2^/m^2^ and showed no statistical differences among BMI class. 94 (33.8%) patients were sarcopenic at the time of initial chemotherapy and the proportion of patients with sarcopenia among BMI class had no significant differences. Cancers were located as follows: 107 (33.8%) intrahepatic bile duct, 67 (24.3%) proximal bile duct (including hilar area), 28 (10.1%) distal bile duct, and 74 (26.8%) gallbladder. Overall, 233 (84.5%) patents were AJCC stage IV at the time of palliative chemotherapy. 44 (15.9%) had an ECOG-PS of 0, 211 (76.4%) had an ECOG-PS of 1, and 22 (7.7%) had an ECOG-PS of 2. Most of the patients (212, 76.8%) received gemcitabine-based palliative chemotherapy. 52 (19.0%) patients received operation before chemotherapy for curative resection or palliative intent and 43 (15.5%) patients received radiotherapy.

**Table 1:** Baseline characteristics of patients.

	All	BMI (kg/m^2^)				p-value
		Underweight	Normal	Overweight	Obese	
		(<18.5)	(18.5–22.9)	(23–24.9)	(≥25)	
		N = 30	N = 113	N = 64	N = 69	
**Age (mean, y)**	63.7 (26–94)	61.3	64.4	64.0	63.5	0.440
**Sex (%)**						<0.001
Male	177 (64.1%)	25 (83.3%)	83 (73.5%)	34 (53.1%)	35 (50.7%)	
Female	99 (35.9%)	5 (16.7%)	30 (26.5%)	30 (46.9%)	34 (49.3%)	
**Location**						0.138
Proximal bile duct (including hilar area)	67 (24.3%)	3 (10.0%)	32 (28.3%)	10 (15.6%)	22 (31.9%)	
Distal bile duct	28 (10.1%)	4 (13.3%)	10 (8.8%)	5 (7.8%)	9 (13.0%)	
Gallbladder	74 (26.8%)	5 (16.7%)	33 (29.2%)	23 (35.9%)	13 (18.8%)	
Intrahepatic bile duct	107 (38.8%)	18 (60.0%)	38 (33.6%)	26 (40.6%)	25 (36.2%)	
**Stage**						0.356
III	43 (15.5%)	3 (10.0%)	11 (9.7%)	11 (17.2%)	17 (25.0%)	
IV	233 (84.5%)	27 (90.0%)	102 (90.3%)	53 (82.8%)	52 (75.0%)	
**OP status**						0.490
No	234 (81.0%)	25 (83.9%)	91 (80.7%)	54 (83.3%)	54 (77.9%)	
Yes	52 (19.0%)	5 (16.1%)	22 (19.3%)	10 (16.7%)	15 (22.1%)	
**Gemcitabine-based chemotherapy**						0.297
No	64 (23.2%)	4 (13.3%)	27 (23.9%)	22 (34.4%)	11 (15.9%)	
Yes	212 (76.8%)	26 (86.7%)	86 (76.1%)	42 (65.6%)	58 (85.1%)	
**ECOG-PS**						0.056
0	44 (15.9%)	6 (20.0%)	12 (10.6%)	14 (21.9%)	12 (17.4%)	
1	211 (76.4%)	20 (66.7%)	88 (77.9%)	48 (75.0%)	55 (79.7%)	
2	22 (7.7%)	4 (13.3%)	13 (11.5%)	2 (3.1%)	2 (2.9%)	
**RT status**						0.123
No	240 (87.0%)	25 (80.6%)	96 (85.0%)	55 (85.9%)	64 (92.8%)	
Yes	36 (13.0%)	5 (19.4%)	17 (15.0%)	9 (14.1%)	5 (7.2%)	
**SMI (cm**^**2**^**/m**^**2**^**)**	43.3±15.5	45.8±14.5	40.5±14.4	45.2±13.1	43.9±19.9	0.294
**Sarcopenia (SMI ≤41 cm**^**2**^**/m**^**2**^**)**						
No	182 (66.2%)	19 (64.5%)	71 (63.0%)	45 (71.2%)	47 (67.6%)	0.594
Yes	94 (33.8%)	11 (35.5%)	42 (37.0%)	19 (28.8%)	22 (32.4%)	

Abbreviations: BMI, body mass index; OP, operation; ECOG-PS, Eastern Cooperative Oncology Group Performance Status; RT, Radiation therapy; SMI. Skeletal muscle index.

Study population was stratified by 2 groups, with age of 64 years and sex then the body weight and composition were analyzed. When divided by the age of 64 years, there was no difference between the two groups in initial value and 2-month value of BMI, and the percentage of body weight change did not differ either. In terms of body composition, the initial value of muscle mass was significantly higher in patients younger than 64 years of age, but there was no statistical significance in 2-month value and rate of change. When analyzed by gender, women had a significantly higher initial BMI than men, but the other indicators did not show any gender differences. (Table 2). And there was no significant differences in the initial SMI, rate of the change and 2-month SMI according to the initial BMI class. (Table 3).

**Table 2:** Change in body weight and composition according to age and sex.

	Initial BMI		2mo BMI		Body weight change (%)	
Age		p = 0.1		p = 0.674		p = 0.296
<64	22.6±3.6		17.4±10.1		-2.98±20.8	
≧64	23.5±4.9		16.8±10.5		-6.11±18.6	
Sex		p<0.001		p = 0.484		p = 0.396
Male	22.4±4.5		16.8±10.5		-4.58±21.0	
Female	24.3±3.8		17.6±9.9		-3.86±16.1	
	Initial SMI		2mo SMI		SMI Change (%)	
Age		p = 0.02		p = 0.005		p = 0.617
<64	45.9±14.1		42.5±12.7		-2.76±7.4	
≧64	40.6±16.4		36.5±16.1		-3.31±7.5	
Sex		p = 0.227		p = 0.58		p = 0.229
Male	44.0±16.2		39.7±15.9		-3.47±7.9	
Female	41.2±13.9		38.4±12.4		-2.17±6.2	

Abbreviations: BMI, body mass index; SMI, skeletal muscle index.

**Table 3:** Initial SMI and its change during 2 month according to BMI class.

	Underweight	Normal	Overweight	Obese	p-value
Initial SMI	45.8±14.5	40.5±14.4	45.2±13.1	43.9±19.9	0.294
2mo SMI	42.0±14.4	36.1±15.1	43.2±11.8	39.3±14.9	0.063
SMI change (%)	-8.56	-9.69	-5.21	-8.96	0.688

Abbreviations: BMI, body mass index; SMI, skeletal muscle index.

### Progression-free survival according to BMI class

**Fig 1:**
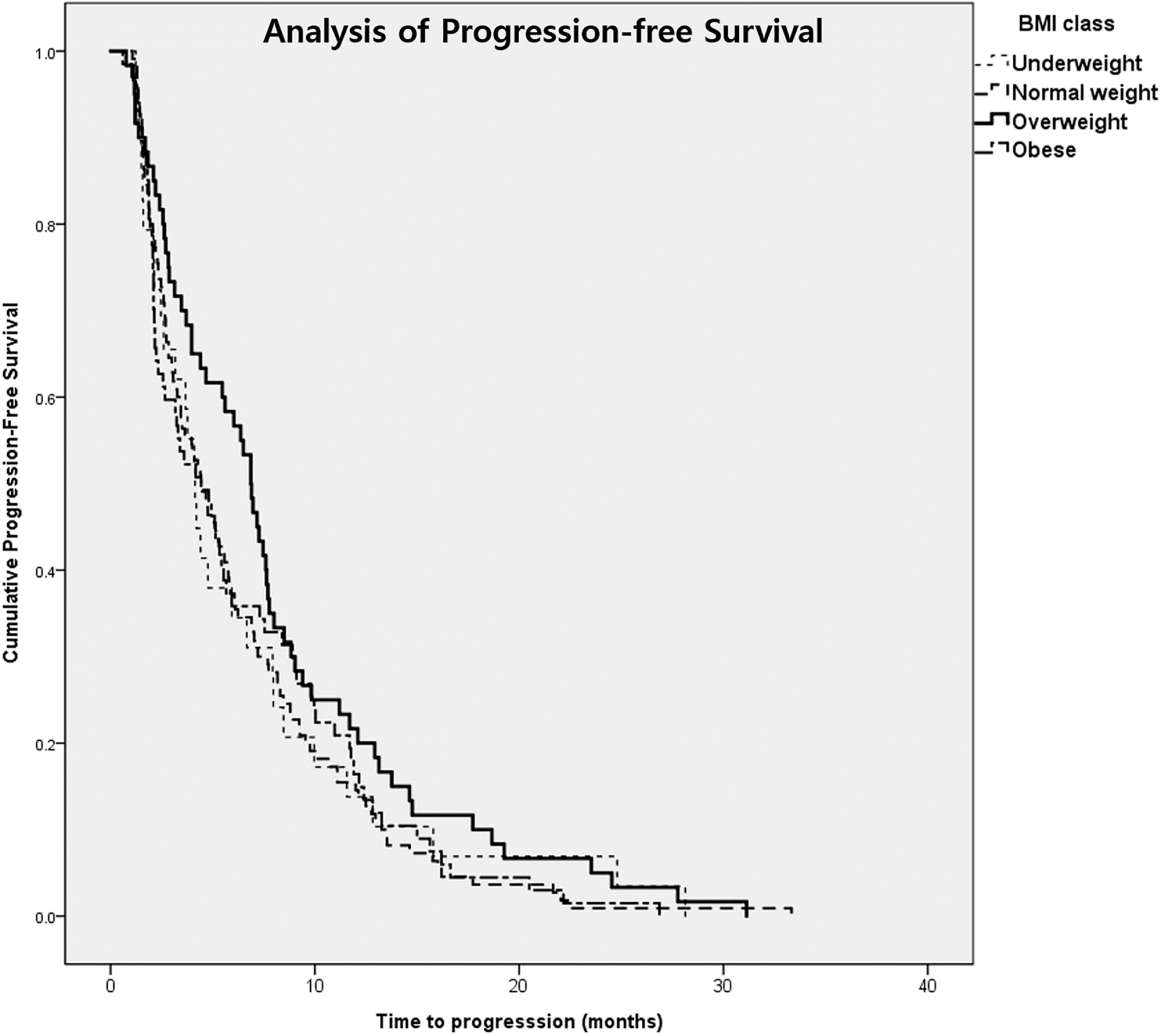
Progression-free survival according to BMI status at baseline.

Overall, 266 patients showed progression of disease during follow-up, and the median PFS was 6.8 months (range: 0.6–33.3, 6.8±6.0). No statistically significant difference in PFS was observed according to BMI. Median PFS was 6.6 months, 6.3 months, 8.1 months and 6.5 months in the normal weight, underweight, overweight, and obese group, respectively (Fig 1). Univariate analysis revealed that non-sarcopenia, gemcitabine-based chemotherapy, stage III, younger age group, and radiotherapy were associated with longer PFS. Upon multivariate analysis, only stage III was independent factor for longer PFS (Table 4).

**Table 4:** Analysis of progression-free survival according to risk factors.

			Univariate		Multivariate	
	N	PFS (median, mo)	Hazard Ratio (95% CI)	p-value	Hazard Ratio (95% CI)	p-value
BMI						
Underweight	110	6.3	1.048 (0.694–1.584)	0.822		
Normal	29	6.6	1			
Overweight	60	8.1	0.797 (0.511–1.243)	0.117		
Obese	67	6.5	1.029 (0.664–1.595)	0.898		
Sarcopenia						
No	182	7.2	1		1	
Yes	84	5.1	1.456 (1.055–2.009)	0.022	1.368 (0.980–1.909)	0.065
Age						
<64	125	7.6	1		1	
≧64	141	6.0	1.295 (1.015–1.651)	0.037	1.106 (0.796–1.535)	0.548
Sex						
Male	175	6.7	1			
Female	91	6.7	0.979 (0.758–1.265)	0.872		
Location						
Proximal bile duct (including hilar area)	66	6.9	1			
Distal bile duct	28	7.8	1.102 (0.533–2.282)	0.793		
Gallbladder	66	6.4	.1.226 (0.793–1.894)	0.360		
Intrahepatic bile duct	106	6.9	0.875 (0.500–1.530)	0.639		
Stage						
3	43	8.4	1		1	
4	223	6.1	1.451 (1.105–1.904)	0.007	1.516 (1.065–2.159)	0.021
OP						
No	216	6.6	1			
Yes	50	8.3	0.861 (0.628–1.180)	0.352		
Gemcitabine						
No	60	5.5	1		1	
Yes	206	7.1	0.741 (0.554–0.991)	0.043	0.795 (0.56–1.128)	0.199
ECOG						
0	42	6.9	1			
1	209	6.4	1.043 (0.854–1.274)	0.591		
2	19	5.9	1.085 (0.630–1.869)	0.77		
RT						
No	231	6.4	1		1	
Yes	35	9.0	0.709 (0.0.495–0.1.016)	0.061	0.788 (0.486–1277)	0.333

Abbreviations: BMI, body mass index; N, number; PFS, progression-free survival; CI, confidence index; Op, operation; ECOG-PS, Eastern Cooperative Oncology Group Performance Status; RT, Radiation therapy.

### Overall survival according to BMI class

**Fig 2:**
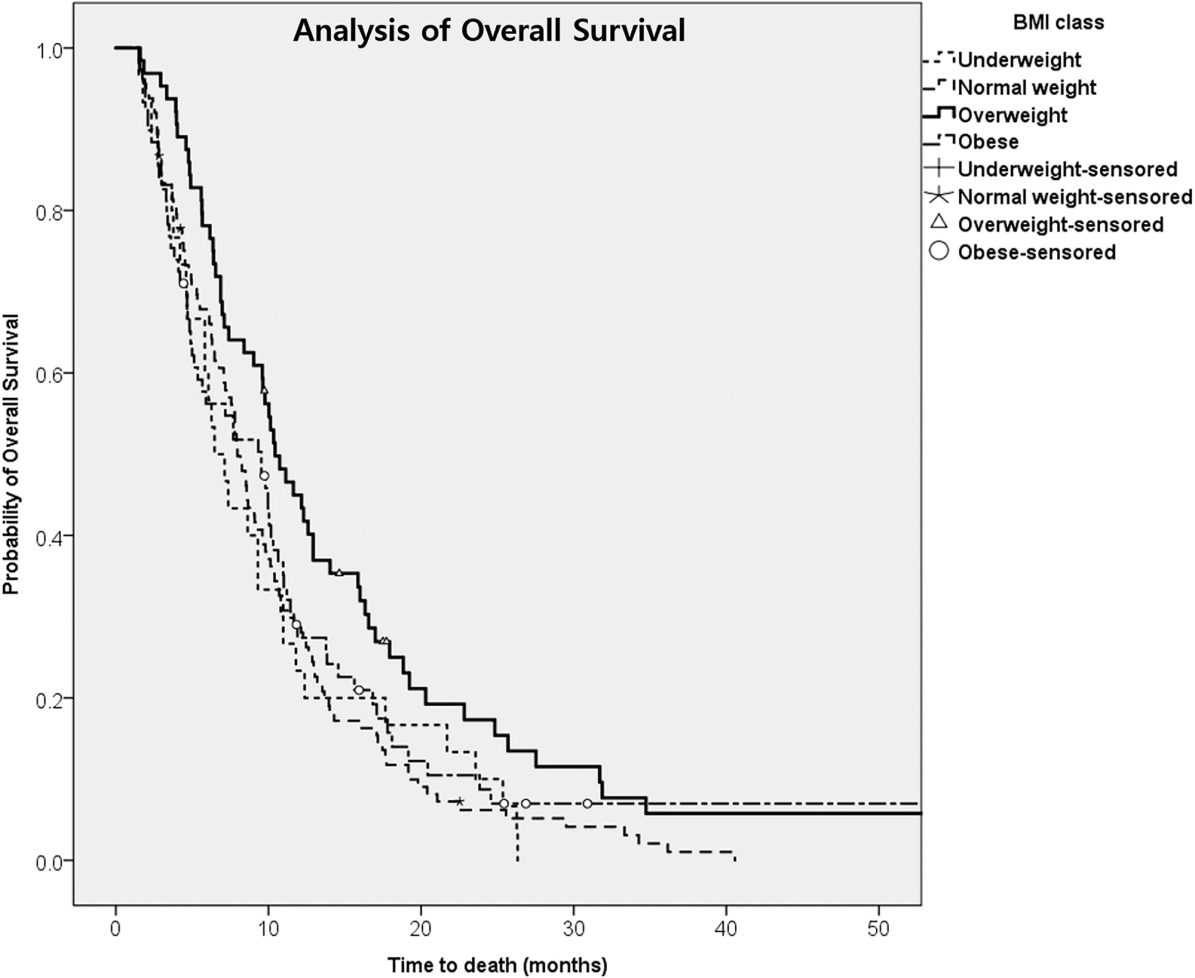
Overall survival according to BMI status at baseline.

A total of 236 deaths or cases of loss of follow-up were recorded in 276 patients. Of the 276 patients, 182 were confirmed to have died and 54 patients had follow-up loss. The mean follow-up period was 11.9 months (1.6–71.6). The median OS was 9.7 months for underweight patients, 10.1 months for normal patients, 15.8 months for the overweight group and 13.1 months for obese patients. Univariate analysis showed that BMI, stage III, age less than 64 year-old, gallbladder cancer, operation, radiotherapy and ECOG performance were significantly associated with better survival. Multivariate analysis was performed and adjusted for age, sex, location, stage, disease status, operation, radiotherapy and ECOG performance. Compared with normal weight patients, overweight patients (BMI 23–24.9kg/m^2^) had a reduced risk of mortality on multivariate analysis (Fig 2, Table 5, HR 0.632; 95% CI 0.436–0.918, p = 0.016) In addition to BMI, tumor location was shown to be associated with overall survival.

**Table 5:** Analysis of overall survival according to risk factors.

			Univariate		Multivariate	
	N	OS (median, mo)	Hazard Ratio (95% CI)	p-value	Hazard Ratio (95% CI)	p-value
BMI						
Underweight	30	9.7	1.044 (0.696–1.567)	0.834	1.096 (0.696–1.728)	0.559
Normal	113	10.1	1		1	
Overweight	64	15.8	0.637 (0.461–0.879)	0.006	0.632 (0.436–0.918)	**0.016**
Obese	69	13.1	0.914 (0.669–1.249)	0.571	1.017 (0.723–1.430)	0.922
Sarcopenia						
No	182	12.0	1	0.378		
Yes	94	10.6	1.152 (0.841–1.579)			
Age						
<64	127	13.3	1		1	
≧64	149	10.2	1.316 (1.028–1.683)	0.029	1.244 (0.934–1.658)	0.135
Sex						
Male	177	11.2	1			
Female	99	12.6	0.831 (0.642–1.074)	0.158		
Location						
Proximal bile duct (including hilar area)	67	9.1	1		1	
Distal bile duct	28	11.6	0.715 (0.453–1.128)	0.149	0.838 (0.504–1.393)	0.495
Gallbladder	74	13.9	0.688 (0.486–0.975)	0.035	0.658 (0.439–0.987)	0.043
Intrahepatic bile duct	107	11.8	0.779 (0.569–1.065)	0.117	0.823 (0.589–1.150)	0.255
Stage						
3	48	14.2	1		1	
4	228	10.4	1.364 (1.051–1.771)	0.02	1.239 (0.926–1.658)	0.150
OP						
No	222	10.4	1		1	
Yes	54	16.3	0.619 (0.450–0.853)	0.003	0.787 (0.544–1.138)	0.203
Gemcitabine						
No	64	12.6	1			
Yes	212	11.4	1.092 (0.820–1.454)	0.546		
ECOG						
0	44	14.1	1		1	
1	211	11.5	1.223 (0.878–1.703)	0.181	1.172 (0.820–1.675)	0.385
2	21	8.6	1.676 (0.982–2.860)	0.058	1.273 (0.676–2.398)	0.455
RT						
No	240	10.9	1		1	
Yes	36	17.1	0.596 (0.411–0.864)	0.006	0.723 (0.474–1.105)	0.134

Abbreviations: BMI, body mass index; N, number; OS, overall survival; CI, confidence index; Op, operation; ECOG-PS, Eastern Cooperative Oncology Group Performance Status; RT, Radiation therapy.

### Overall survival according to change in body weight, composition and BMI

**Fig 3:**
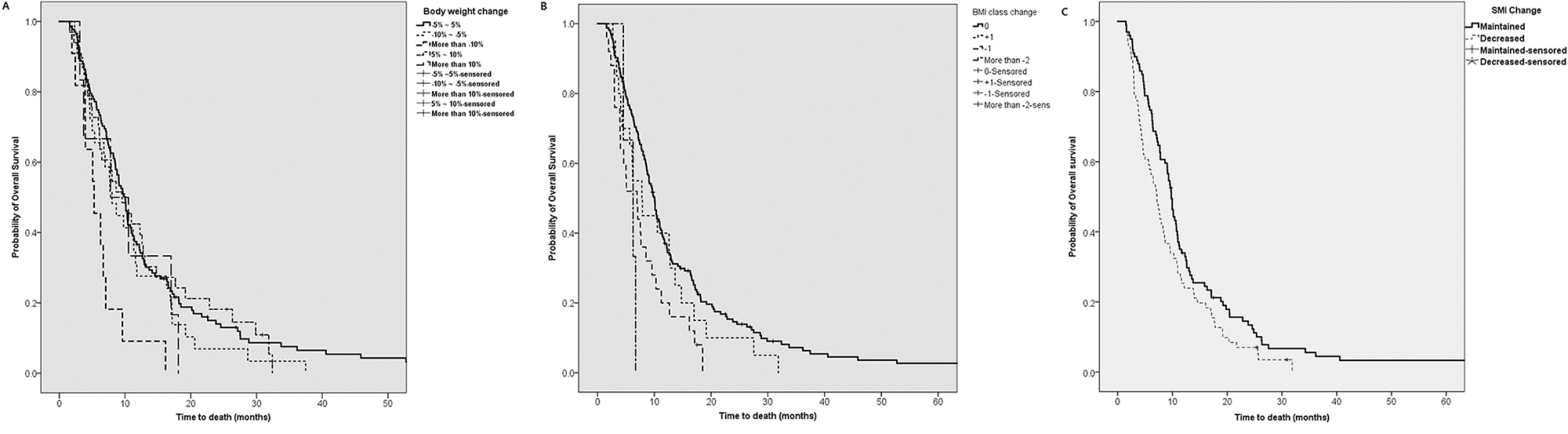
Overall survival according to change of body weight (A), BMI class (B) and SMI (C) during the first two months of chemotherapy.

We conducted additional analyses to determine if changes in body weight, BMI and body composition affected overall survival. The median overall survival was longest in the group of no change, while the group in which body weight decreased by more than 10% showed the worst prognosis (Table 6, Fig 3A). Survival analysis of the BMI class revealed a statistically significant decrease in groups in which BMI class had decreased. Overall survival was longest in the group without change of BMI class (Table 6, Fig 3B). Greater changes in body weight were associated with less overall survival.

**Table 6:** Overall survival according to change in body weight, BMI class and SMI during the first two months of chemotherapy.

			Univariate		Multivariate	
	N	OS (median, mo)	Hazard Ratio (95% CI)	p-value	Hazard Ratio (95% CI)	p-value
Body weight change						
-5% to 5%	173	13.7	1		1	
-10% to -5%	38	9.1	1.428 (0.947–2.154)	0.89	1.076 (0.651–1.778)	0.776
more than -10%	14	5.8	2.686 (1.394–5.175)	0.003	1.757 (0.670–4.610)	0.252
5% to 10%	43	11.9	1.000 (0.663–1.507)	0.999	0.860 (0.491–1.504)	0.860
more than 10%	8	9.9	1.283 (0.563–2.923)	0.554	1.135 (0.407–3.164)	0.809
BMI class change						
0	214	12.6	1		1	
+1	26	13.8	0.870 (0.572–1.323)	0.470	0.931 (0.730–1.780)	0.436
-1	33	11.7	1.062 (0.743–1.519)	0.740	1.252 (0.680–2.306)	0.471
more than -2	3	8.4	1.406 (1.049–1.691)	0.041	1.410 (1.168–1.986)	0.046
SMI change						
Maintained	157	13.4	1		1	
Decreased	119	9.4	1.410 (1.029–1.932)	0.033	1.391 (1.015–1.907)	0.04

Abbreviations: N, number; OS, overall survival; BMI, body mass index; SMI, skeletal muscle index.

During the initial 2 months, 119 (41.9%) patients suffered decreased SMI by more than 10%. The overall survival of maintained SMI group was 13.4 months and 9.4 months in the decreased SMI group, indicating statistically significant decrease in the survival time in the decreased SMI group. Deceased SMI was associated with poor overall survival. (Table 6, Fig 3C.)

We evaluated the overall survival according to initial muscle mass and change in muscle mass in each BMI group. Overall survival was not different significantly according to initial sarcopenia or decrease in muscle mass during 2 months among underweight, normal weight, and obese groups. In the overweight group, the overall survival of the initial sarcopenic patients was significantly lower than that of the non-sarcopenic group. The decrease in muscle mass did not significantly affect overall survival. (Table 7)

**Table 7:** Overall survival according to body composition and its change in each BMI group.

	Initial SMI			SMI change		
BMI	Sarcopenia	Non-sarcopenia	p-value	Maintained	Decreased	p-value
Underweight	7.58	10.7	0.226	12.2	7.8	0.105
Normal	7.6	10.9	0.062	10.8	8.3	0.199
Overweight	11.5	22.2	0.028	16.6	11.6	0.5
Obese	8.4	17.1	0.101	15.2	10.5	0.594

Abbreviations: SMI, skeletal muscle index; BMI, body mass index.

## Discussion

Although the obesity paradox has been recognized in many kinds of cancer, the association between weight status and the prognosis of biliary tract cancer patients has seldom been reported. Several studies have demonstrated an association between obesity and risk of biliary tract cancer incidence, but none have investigated prognosis of biliary tract cancer according to weight status to the best of our knowledge. This is the first report that showed overweight status can prognosticate better survival in patients with advanced biliary tract cancer who received palliative chemotherapy.

In this study, survival outcome was better in the overweight group than other groups. These findings are consistent with the results of other previous cancer studies that examined the relationship between obesity and survival outcome. In many studies, the pattern of survival outcome was similar to that of U shape [14], which is concordant with the results of the present study. It is worth of noting that BMI at the onset of chemotherapy is associated with overall survival, independent of skeletal muscle mass.

As clinical/biological markers that can reflect cachexia/proteolysis, change in skeletal muscle mass have been focused on. In previous studies, sarcopenia at diagnosis and decrease in muscle mass during clinical course were known to have association with poor overall survival in patients with advanced cancer [11, 15, 16]. In this study, there was a significant correlation between the maintenance of muscle mass and overall survival as previous studies. Significant decrease in muscle mass was associated with poor overall survival but initial muscle mass itself did not affect significant difference in overall survival. Preceding studies were mainly based on the western population, which suggests that current threshold for sarcopenia may not fit well with the Asian population. There was a study that the cutoff value for sarcopenia were set arbitrarily by ROC analysis in Asian population [16], but more studies would be needed for validation. Skeletal muscle mass index has been known as a useful indicator for determining sarcopenia in several studies, but the most adequate method and its threshold for sarcopenia are still under debate [13, 17]. Although not statistically significant, this study showed that overall survival has tendency to decrease in sarcopenic patients and decrease in overall muscle mass, independent of initial muscle mass, was associated with decrease in overall survival and those results are in line with other studies.

There are several possible reasons for the relatively good prognosis of the overweight group. One potential explanation is that fat can act as the main energy reservoir of the body; therefore, an adequate amount of body fat confers a survival advantage in cancer patients [18]. Another possibility is that BMI is not an accurate measure of body adiposity and composition, and cannot distinguish lean body mass from fat mass. Indeed, muscular individuals could be included in the overweight group during classification through BMI [19]. Finally, the low-body weight group may have a higher mean age, more comorbidities, and a lower performance status than the other groups. Thus, older age and comorbidities may act as possible confounding factors when attempting to determine the association between BMI categories and survival outcome in cancer patients [20, 21].

The poor survival outcome of the underweight group may be related to the loss of muscle and fat mass because of sarcopenia and cachexia. Several studies also demonstrated that sarcopenia has been found to be associated with poor overall prognosis in patients with cancer [15, 22–25]. Cancer-induced cachexia is known to be not fully reversible by usual nutritional support, leading to progressive functional impairment, reduced tolerance to treatment, and finally decreased survival rates [19, 26, 27].

In contrast to overweight, obesity was not associated with longer survival in patients with cancer in advanced stages. There is also a high possibility that co-morbidities, especially those associated with metabolic diseases, could increase. The increase in excess visceral adipose tissue is associated with an increase in interleukin-6 (IL-6), free fatty acid, and tumor necrosis factor-alpha (TNF-a). This increase in cytokine causes increases in insulin resistance and pro-angiogenic factors, and promotes chronic inflammatory state leading to development of the tumor microenvironment [28]. This may be the cause of development of chemo-resistance and poor response to chemotherapy. In general, when chemotherapy is given, patients with a BSA greater than 2 are more likely to undergo dose-capping with BSA 2 for fear of toxicity. These unnecessary reductions in chemotherapy dosing may have resulted in poor treatment results among obese patients compared to overweight patients [29].

As the degree of weight change in the first 2 months of chemotherapy increased, the overall survival tended to decrease. Previous studies of other carcinomas have reported that patient weight loss has an adverse effect on clinical outcome [30], which is concordant with the results of the present study. Weight loss in the early period of chemotherapy is known to occur in about 85% of advanced cancer patients [31], and tumor-induced hyper-metabolism [32] may be the first cause of the weight loss. It is possible that increased tumor burden due to cancer progression resulted in loss of weight and decreased overall survival. In addition, as tumors with high malignant potential require more energy for growth, it is possible that aggressive biologic tumor behavior is expressed in the form of weight loss.

To evaluate whether tumor biology has significant effects in the initial nutritional status and the progression of cachexia, we compared the absolute value of SMI and the ratio of sarcopenia in each BMI class at the beginning of chemotherapy and 2 month later. No significant differences were found in the aspect of muscle mass. Therefore, we assumed that BMI can be one of the predictors of overall survival independent of change in muscle mass.

There was no significant association between BMI category and progression-free survival. Progression of the tumor may primarily be due to factors such as tumor burden which is represented by stage, biology and chemo-resistance rather than host factors, as reported for other types of malignancies. However, significant differences in the final outcome according to the BMI category indicate the importance of host factors such as infection control, adequate nutritional status and its maintenance, which can be achieved by adequate supportive care during treatment.

One major limitation of our study is that it was retrospective, as were previous studies that investigated the association between BMI and survival outcome in other cancers. Accordingly, it may be difficult to apply these results to the general population because they are based on a single tertiary center and the criteria for overweight and obese groups were applied slightly differently because of ethnicity. Furthermore, because we only evaluated the BMI at the time of initiation of chemotherapy, the results could not reflect the changes in BMI during the long-term follow-up period and its impact. Another limitation is that BMI is not an accurate measure of body adiposity and composition as described above. Combining tools such as waist circumference, waist-to-hip ratio, and skinfold with BMI may be better for evaluating body components. Such analyses can be accomplished by CT, MRI, and dual-energy X-ray absortiometry. Overall, further efforts are needed to distinguish between adipose tissue (SAT) and visceral adipose tissue (VAT).

Nevertheless, to the best of our knowledge, this is the first study to demonstrate the association of BMI, body weight change and prognosis in patients with advanced BTC. The results presented herein indicate that evaluation of nutritional status in patients during chemotherapy is important. Therefore, clinicians should pay attention to the weight and nutritional status of patients at the time of chemotherapy and provide supportive care to maintain adequate nutritional status. Moreover, there is the possibility to improve clinical outcome by more aggressive chemotherapy in specific groups according to BMI.

## Conclusion

Overweight status at the time of chemotherapy and maintenance of body weight during the initial period of chemotherapy are important prognostic factors of better overall survival for patients with advanced biliary tract cancers.

## Supporting information

S1 File Dataset for body mass index and skeletal muscle index. (ZIP)

## References

[1] Esnaola NF, Meyer JE, Karachristos A, Maranki JL, Camp ER, Denlinger CS. Evaluation and management of intrahepatic and extrahepatic cholangiocarcinoma. Cancer. 2016;122(9):1349–69. Epub 2016/01/23. doi: 10.1002/cncr.29692 . 26799932 10.1002/cncr.29692

[2] Myers J, Lata K, Chowdhury S, McAuley P, Jain N, Froelicher V. The obesity paradox and weight loss. The American journal of medicine. 2011;124(10):924–30. Epub 2011/07/30. doi: 10.1016/j.amjmed.2011.04.018 . 21798508 10.1016/j.amjmed.2011.04.018

[3] Kasenda B, Bass A, Koeberle D, Pestalozzi B, Borner M, Herrmann R, et al Survival in overweight patients with advanced pancreatic carcinoma: a multicentre cohort study. BMC cancer. 2014;14:728 Epub 2014/10/01. doi: 10.1186/1471-2407-14-728 mc4242603. 25266049 10.1186/1471-2407-14-728 PMC4242603

[4] Tsang NM, Pai PC, Chuang CC, Chuang WC, Tseng CK, Chang KP, et al Overweight and obesity predict better overall survival rates in cancer patients with distant metastases. Cancer medicine. 2016;5(4):665–75. Epub 2016/01/27. doi: 10.1002/cam4.634 . 26811258 10.1002/cam4.634 PMC4831285

[5] Li JS, Han TJ, Jing N, Li L, Zhang XH, Ma FZ, et al Obesity and the risk of cholangiocarcinoma: a meta-analysis. Tumour biology: the journal of the International Society for Oncodevelopmental Biology and Medicine. 2014;35(7):6831–8. Epub 2014/04/15. doi: 10.1007/s13277-014-1939-4 . 24729126 10.1007/s13277-014-1939-4

[6] Li ZM, Wu ZX, Han B, Mao YQ, Chen HL, Han SF, et al The association between BMI and gallbladder cancer risk: a meta-analysis. Oncotarget. 2016 Epub 2016/06/02. doi: 10.18632/oncotarget.9664 . 27248320 10.18632/oncotarget.9664 PMC5190051

[7] Tan W, Gao M, Liu N, Zhang G, Xu T, Cui W. Body Mass Index and Risk of Gallbladder Cancer: Systematic Review and Meta-Analysis of Observational Studies. Nutrients. 2015;7(10):8321–34. Epub 2015/10/02. doi: 10.3390/nu7105387 . 26426043 10.3390/nu7105387 PMC4632410

[8] Calle EE, Rodriguez C, Walker-Thurmond K, Thun MJ. Overweight, obesity, and mortality from cancer in a prospectively studied cohort of U.S. adults. The New England journal of medicine. 2003;348(17):1625–38. Epub 2003/04/25. doi: 10.1056/NEJMoa021423 . 12711737 10.1056/NEJMoa021423

[9] Farhat MH, Shamseddine AI, Tawil AN, Berjawi G, Sidani C, Shamseddeen W, et al Prognostic factors in patients with advanced cholangiocarcinoma: role of surgery, chemotherapy and body mass index. World journal of gastroenterology. 2008;14(20):3224–30. Epub 2008/05/29. doi: 10.3748/wjg.14.3224 mc2712857. 18506930 10.3748/wjg.14.3224 PMC2712857

[10] Mitsiopoulos N, Baumgartner RN, Heymsfield SB, Lyons W, Gallagher D, Ross R. Cadaver validation of skeletal muscle measurement by magnetic resonance imaging and computerized tomography. Journal of applied physiology (Bethesda, Md: 1985). 1998;85(1):115–22. Epub 1998/07/09. doi: 10.1152/jappl.1998.85.1.115 . 9655763 10.1152/jappl.1998.85.1.115

[11] Martin L, Birdsell L, Macdonald N, Reiman T, Clandinin MT, McCargar LJ, et al Cancer cachexia in the age of obesity: skeletal muscle depletion is a powerful prognostic factor, independent of body mass index. Journal of clinical oncology: official journal of the American Society of Clinical Oncology. 2013;31(12):1539–47. Epub 2013/03/27. doi: 10.1200/jco.2012.45.2722 . 23530101 10.1200/JCO.2012.45.2722

[12] Kim MK, Lee W-Y, Kang J-H, Kang J-H, Kim BT, Kim SM, et al 2014 Clinical Practice Guidelines for Overweight and Obesity in Korea. Endocrinol Metab. 2014;29(4):405–9. 10.3803/EnM.2014.29.4.405 PMC4285036 25559568

[13] Shachar SS, Deal AM, Weinberg M, Nyrop KA, Williams GR, Nishijima TF, et al Skeletal Muscle Measures as Predictors of Toxicity, Hospitalization, and Survival in Patients with Metastatic Breast Cancer Receiving Taxane-Based Chemotherapy. Clinical cancer research: an official journal of the American Association for Cancer Research. 2017;23(3):658–65. Epub 2016/08/05. doi: 10.1158/1078-0432.ccr-16-0940 . 27489287 10.1158/1078-0432.CCR-16-0940 PMC5290138

[14] Lennon H, Sperrin M, Badrick E, Renehan AG. The Obesity Paradox in Cancer: a Review. Current oncology reports. 2016;18(9):56 Epub 2016/08/01. doi: 10.1007/s11912-016-0539-4 . 27475805 10.1007/s11912-016-0539-4 PMC4967417

[15] Meza-Junco J, Montano-Loza AJ, Baracos VE, Prado CM, Bain VG, Beaumont C, et al Sarcopenia as a prognostic index of nutritional status in concurrent cirrhosis and hepatocellular carcinoma. Journal of clinical gastroenterology. 2013;47(10):861–70. Epub 2013/06/12. doi: 10.1097/MCG.0b013e318293a825 . 23751844 10.1097/MCG.0b013e318293a825

[16] Choi Y, Oh DY, Kim TY, Lee KH, Han SW, Im SA, et al Skeletal Muscle Depletion Predicts the Prognosis of Patients with Advanced Pancreatic Cancer Undergoing Palliative Chemotherapy, Independent of Body Mass Index. PloS one. 2015;10(10):e0139749 Epub 2015/10/06. doi: 10.1371/journal.pone.0139749 . 26437072 10.1371/journal.pone.0139749 PMC4593598

[17] Rutten IJG, Ubachs J, Kruitwagen R, Beets-Tan RGH, Olde Damink SWM, Van Gorp T. Psoas muscle area is not representative of total skeletal muscle area in the assessment of sarcopenia in ovarian cancer. Journal of cachexia, sarcopenia and muscle. 2017;8(4):630–8. Epub 2017/05/18. doi: 10.1002/jcsm.12180 . 28513088 10.1002/jcsm.12180 PMC5566632

[18] Bouillanne O, Dupont-Belmont C, Hay P, Hamon-Vilcot B, Cynober L, Aussel C. Fat mass protects hospitalized elderly persons against morbidity and mortality. The American journal of clinical nutrition. 2009;90(3):505–10. Epub 2009/07/31. doi: 10.3945/ajcn.2009.27819 . 19640947 10.3945/ajcn.2009.27819

[19] Gonzalez MC, Pastore CA, Orlandi SP, Heymsfield SB. Obesity paradox in cancer: new insights provided by body composition. The American journal of clinical nutrition. 2014;99(5):999–1005. Epub 2014/02/28. doi: 10.3945/ajcn.113.071399 . 24572565 10.3945/ajcn.113.071399

[20] Hines RB, Shanmugam C, Waterbor JW, McGwin G Jr., Funkhouser E, Coffey CS, et al Effect of comorbidity and body mass index on the survival of African-American and Caucasian patients with colon cancer. Cancer. 2009;115(24):5798–806. Epub 2009/11/26. doi: 10.1002/cncr.24598 . 19937953 10.1002/cncr.24598 PMC2795032

[21] Banack HR, Kaufman JS. From bad to worse: collider stratification amplifies confounding bias in the "obesity paradox". European journal of epidemiology. 2015;30(10):1111–4. Epub 2015/07/19. doi: 10.1007/s10654-015-0069-7 . 26187718 10.1007/s10654-015-0069-7

[22] Kaneko M, Sasaki S, Ozaki K, Ishimaru K, Terai E, Nakayama H, et al Underweight status predicts a poor prognosis in elderly patients with colorectal cancer. Molecular and clinical oncology. 2016;5(3):289–94. Epub 2016/09/08. doi: 10.3892/mco.2016.964 . 27602223 10.3892/mco.2016.964 PMC4998352

[23] Janssen I, Heymsfield SB, Ross R. Low relative skeletal muscle mass (sarcopenia) in older persons is associated with functional impairment and physical disability. Journal of the American Geriatrics Society. 2002;50(5):889–96. Epub 2002/05/25. . 12028177 10.1046/j.1532-5415.2002.50216.x

[24] Sabel MS, Lee J, Cai S, Englesbe MJ, Holcombe S, Wang S. Sarcopenia as a prognostic factor among patients with stage III melanoma. Annals of surgical oncology. 2011;18(13):3579–85. Epub 2011/08/09. doi: 10.1245/s10434-011-1976-9 . 21822551 10.1245/s10434-011-1976-9

[25] Lanic H, Kraut-Tauzia J, Modzelewski R, Clatot F, Mareschal S, Picquenot JM, et al Sarcopenia is an independent prognostic factor in elderly patients with diffuse large B-cell lymphoma treated with immunochemotherapy. Leukemia & lymphoma. 2014;55(4):817–23. Epub 2013/06/21. doi: 10.3109/10428194.2013.816421 . 23781925 10.3109/10428194.2013.816421

[26] Fouladiun M, Korner U, Bosaeus I, Daneryd P, Hyltander A, Lundholm KG. Body composition and time course changes in regional distribution of fat and lean tissue in unselected cancer patients on palliative care—correlations with food intake, metabolism, exercise capacity, and hormones. Cancer. 2005;103(10):2189–98. Epub 2005/04/12. doi: 10.1002/cncr.21013 . 15822132 10.1002/cncr.21013

[27] Murphy RA, Wilke MS, Perrine M, Pawlowicz M, Mourtzakis M, Lieffers JR, et al Loss of adipose tissue and plasma phospholipids: relationship to survival in advanced cancer patients. Clinical nutrition (Edinburgh, Scotland). 2010;29(4):482–7. Epub 2009/12/05. doi: 10.1016/j.clnu.2009.11.006 . 19959263 10.1016/j.clnu.2009.11.006

[28] Arnold M, Leitzmann M, Freisling H, Bray F, Romieu I, Renehan A, et al Obesity and cancer: An update of the global impact. Cancer epidemiology. 2016;41:8–15. Epub 2016/01/18. doi: 10.1016/j.canep.2016.01.003 . 26775081 10.1016/j.canep.2016.01.003

[29] Horowitz NS, Wright AA. Impact of obesity on chemotherapy management and outcomes in women with gynecologic malignancies. Gynecologic oncology. 2015;138(1):201–6. Epub 2015/04/15. doi: 10.1016/j.ygyno.2015.04.002 . 25870918 10.1016/j.ygyno.2015.04.002 PMC4469517

[30] Yildirim BA, Ozdemir Y, Colakoglu T, Topkan E. Impact of presence and degree of pretreatment weight loss in locally-advanced pancreatic cancer patients treated with definitive concurrent chemoradiotherapy. Pancreatology: official journal of the International Association of Pancreatology (IAP) [et al]. 2016;16(4):599–604. Epub 2016/04/01. doi: 10.1016/j.pan.2016.03.006 . 27029854 10.1016/j.pan.2016.03.006

[31] Barber MD, Ross JA, Fearon KCH. Changes in Nutritional, Functional, and Inflammatory Markers in Advanced Pancreatic Cancer. Nutrition and Cancer. 1999;35(2):106–10. doi: 10.1207/S15327914NC352_2 10693162 10.1207/S15327914NC352_2

[32] Wigmore SJ, Todorov PT, Barber MD, Ross JA, Tisdale MJ, Fearon KCH. Characteristics of patients with pancreatic cancer expressing a novel cancer cachectic factor. British Journal of Surgery. 2000;87(1):53–8. 10606911 10.1046/j.1365-2168.2000.01317.x

